# Functional Protein Network Activation Mapping Reveals New Potential Molecular Drug Targets for Poor Prognosis Pediatric BCP-ALL

**DOI:** 10.1371/journal.pone.0013552

**Published:** 2010-10-21

**Authors:** Benedetta Accordi, Virginia Espina, Marco Giordan, Amy VanMeter, Gloria Milani, Luisa Galla, Maria Ruzzene, Manuela Sciro, Luca Trentin, Ruggero De Maria, Geertruy te Kronnie, Emanuel Petricoin, Lance Liotta, Giuseppe Basso

**Affiliations:** 1 Oncohematology Laboratory, Department of Pediatrics, University of Padova, Padova, Italy; 2 Center for Applied Proteomics and Molecular Medicine, George Mason University, Manassas, Virginia, United States of America; 3 Department of Biological Chemistry and Venetian Institute of Molecular Medicine (VIMM), University of Padova, Padova, Italy; 4 Department of Hematology, Oncology and Molecular Medicine, Istituto Superiore di Sanità, Roma, Italy; University of Minnesota, United States of America

## Abstract

**Background:**

In spite of leukemia therapy improvements obtained over the last decades, therapy is not yet effective in all cases. Current approaches in Acute Lymphoblastic Leukemia (ALL) research focus on identifying new molecular targets to improve outcome for patients with a dismal prognosis. In this light phosphoproteomics seems to hold great promise for the identification of proteins suitable for targeted therapy.

**Methodology/Principal Findings:**

We employed Reverse Phase Protein Microarrays to identify aberrantly activated proteins in 118 pediatric B-cell precursor (BCP)-ALL patients. Signal transduction pathways were assayed for activation/expression status of 92 key signalling proteins. We observed an increased activation/expression of several pathways involved in cell proliferation in poor clinical prognosis patients. MLL-rearranged tumours revealed BCL-2 hyperphosphorylation through AMPK activation, which indicates that AMPK could provide a functional role in inhibiting apoptosis in MLL-rearranged patients, and could be considered as a new potential therapeutic target. Second, in patients with poor clinical response to prednisone we observed the up-modulation of LCK activity with respect to patients with good response. This tyrosine-kinase can be down-modulated with clinically used inhibitors, thus modulating LCK activity could be considered for further studies as a new additional therapy for prednisone-resistant patients. Further we also found an association between high levels of CYCLIN E and relapse incidence. Moreover, CYCLIN E is more expressed in early relapsed patients, who usually show an unfavourable prognosis.

**Conclusions/Significance:**

We conclude that functional protein pathway activation mapping revealed specific deranged signalling networks in BCP-ALL that could be potentially modulated to produce a better clinical outcome for patients resistant to standard-of-care therapies.

## Introduction

Acute Lymphoblastic Leukemia (ALL) is the most common form of pediatric cancer with a worldwide incidence of about 1–4.75 per 100 000 persons [1]. Remarkable progress has been made in treatment of childhood ALL but therapy is not yet effective in all cases. Current research interest focuses on identifying new specific molecular drug targets for new patient-tailored approaches that can improve therapy efficacy and reduce toxicity. Knowledge of deregulation of cell signalling pathways in cancer that regulate and control cell proliferation, differentiation, survival and death forms the basis for understanding tumour progression. Recent publications have placed elucidation of protein signalling pathways at the central point in the effective treatment of cancer [2], [3]. Pathway activation and function is controlled by post-translational modifications, mainly by phosphorylation, underpinned by ongoing activity of protein kinases and phosphatases. Consequently, functional pathway mapping technology that can directly measure the activation state of hundreds of proteins in signalling transduction pathways (STPs), can hold great promise for the identification of altered STPs in tumour cells. Such efforts promise to potentially provide new targets for rational, molecular-targeted drug design and could identify cancer patients that may benefit from the use of specific targeted inhibitors [4], [5]. Protein activation status can not be directly analyzed through gene expression profiling, since post-translational modifications, such as phosphorylation, are not predictable from gene expression levels. Here, Reverse Phase Protein Microarray (RPMA) technology had been used to profile the activation state of 92 key molecules in a cohort of 118 newly diagnosed pediatric BCP-ALL patients, in order to identify and map pathway activation changes associated with clinical characteristics. This innovative technique can measure the activation levels/phosphorylation of large numbers of signalling proteins at once from small clinical samples in a very reproducible, precise, sensitive and high-throughput manner. The RPMA format immobilizes in spots dozens of different patient samples on one array and each array is then incubated with a specific antibody, thus a single endpoint is measured and directly compared across multiple samples without introduction of experimental variability. This cutting-edge technology has already been applied with success to profile the cellular STPs activity in several cancers [4]–[9]. We observed an increase or decrease in activation/phosphorylation state of signalling proteins within specific protein networks in clinical poor prognosis patients cohorts. In particular, here we show the inhibition of the LCK kinase in Prednisone Good Responder (PGR) patients, and a hyperactivated pathway in the MLL-rearranged cohort of patients that leads to BCL-2 activation through LKB1 and AMPK phosphorylation. Moreover, we found a correlation between CYCLIN E expression and Relapse Free Survival (RFS) rates: patients who show high levels of CYCLIN E expression have a more elevated probability to relapse. These new information on pediatric BCP-ALL activated protein patterns provided by phosphoproteomic analyses with RPMA will be the start for future functional studies with specific protein inhibitors, in order to point out new drugs for patient tailored therapies.

## Results

### Correlation between Protein Expression and Clinical Characteristics

We first searched for correlation between protein expression/activation and patients clinical characteristics. In particular we considered the followings: age (1–9 years vs >9 years), sex, white blood cell count (WBC > vs < of 50×10^9^/L), DNA index (1–1.15 vs ≥1.16), chromosomal translocations (non-translocated, t(9;22), t(12;21), t(1;19) and MLL rearrangements), Minimal Residual Disease (Low Risk, Medium Risk, High Risk), immunophenotype (Prepre-B, Pre-B, CALL, Prepre-B/CALL) and prednisone response through Wilcoxon tests or two-sample Welch t-tests implemented in multtest package. No correlation was found between protein expression/activation and age, sex, WBC, DNA index, Minimal Residual Disease (MRD) and immunophenotype (data not shown), but we observed differentially activated/expressed proteins in MLL-rearranged vs non-translocated and in Prednisone Good Responder (PGR) vs Prednisone Poor Responder (PPR) patients comparisons.

### AMPK Pathway is Hyperactivated in MLL-rearranged Patients

**Figure 1:**
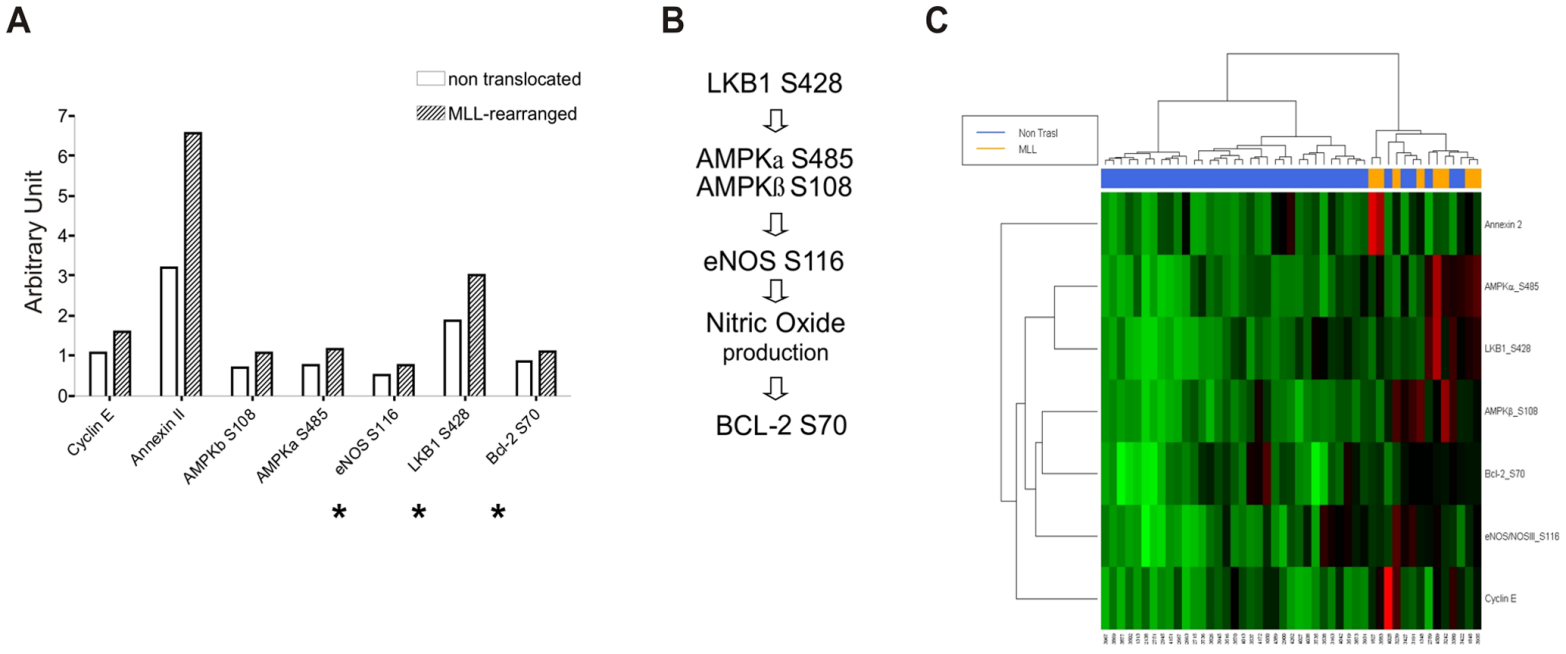
Hyperactivation of the AMPK pathway in MLL-rearranged patients. (A) Histogram of proteins, that are part of the AMPK pathway, found to be differentially activated (Wilcoxon test with Benjamini-Hochberg multiplicity correction, p<0.05) between the MLL- and non-translocated cohorts. * indicates proteins that are part of the AMPK pathway, but that did reach statistical significance using Global Test analysis (p = 0.003). (B) Scheme of the AMPK pathway. (C) Heatmap with hierarchical clustering. The heatmap was generated with R Project using the proteins differentially expressed/phosphorylated in the “MLL-rearranged patients” group vs “non-translocated patients” group comparison. MLL-rearranged patients are highlighted in orange.

**Figure 2:**
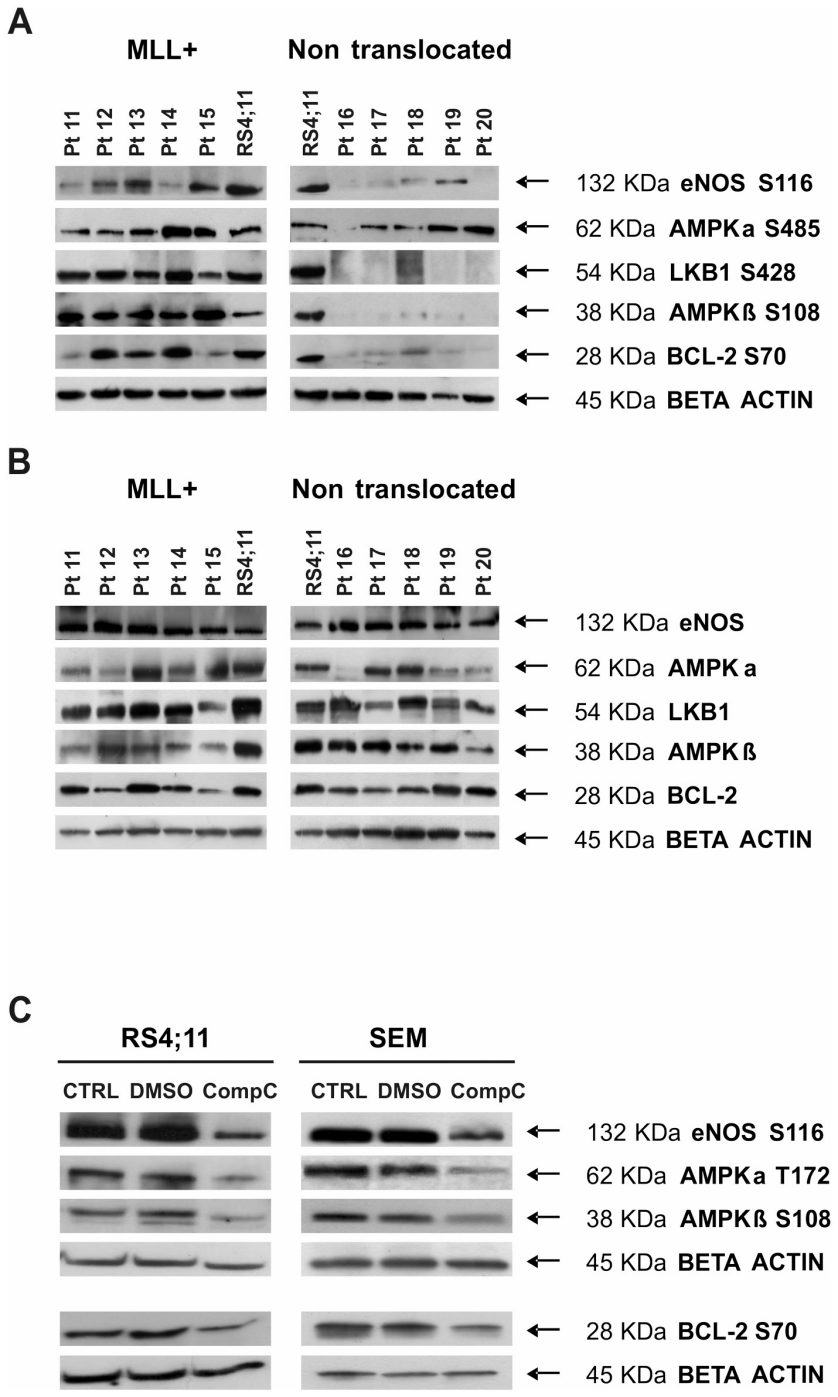
Validation of RPMA results through Western Blot. (A) Hyperactivation of the AMPK pathway in MLL-rearranged patients vs non-translocated ones (independent sets of pediatric BCP-ALL at diagnosis: patients 11–15 are MLL-rearranged -all MLL-AF4-, patients 16–20 are non-translocated). RS4;11 cell lysate was used as positive control for antibody staining. (B) Total forms of the AMPK pathway proteins in previously described patients: 11–15 are MLL-rearranged and 16–20 are non-rearranged. RS4;11 cell lysate was used as positive control for antibody staining. There are no substantial differences on total protein form levels between MLL-rearranged and non-translocated patients. (C) AMPK pathway inhibition after Compound C treatment. RS4;11 and SEM cells (both MLL-rearranged) were treated with the AMPK inhibitor Compound C 8 µM for 48 hours. Phosphorylation of AMPK pathway proteins was evaluated through WB in control, DMSO treated and Compound C treated cells.

We compared primary leukemia samples isolated from 8 MLL-rearranged patients (5 with t(4;11), 2 with t(9;11), and one with t(11;19)) with 36 patients without known genomic aberrancies. Statistical analysis (Wilcoxon test with Benjamini-Hochberg multiplicity corrections) revealed different expression or activation of 9 proteins between the MLL-rearranged patients and the non-translocated ones. Our results show that 4 proteins were statistically significantly elevated in the MLL-rearranged patients group: CYCLIN E (p = 0.02425), ANNEXIN 2 (p = 0.02910), AMPKβ (S108) (p = 0.02910) and AMPKα (S485) (p = 0.03686). Furthermore a set of 3 more proteins, eNOS/NOS III (S116 – corresponding to S114 in human), LKB1 (S428) and BCL-2 (S70), was found to be differentially activated in the MLL-rearranged cohort using a Global Test analysis (p = 0.003) (**Figure 1A**). These 3 proteins are all known members of the AMPK pathway (see Discussion) and, along with the findings that AMPK itself was activated in the MLL-rearranged cohort, form the basis of a comprehensive pathway derangement in MLL-rearranged patients (presented schematically in **Figure 1B**). We thus identified a singular MLL-specific hyperactivated pathway that through AMPK phosphorylation leads to the activation of BCL-2. A heatmap was generated to highlight the relationships between clustering and protein expression levels (**Figure 1C**). RPMA results were validated by Western Blot in an independent set of patients (**Figure 2A**). Of note, total forms of the AMPK pathway proteins do not show substantial differences between MLL-rearranged and non-translocated patients (**Figure 2B**), corroborating the observation that the higher phosphorylation levels of the proteins in the AMPK pathway are the peculiar molecular derangement characteristic of MLL-rearranged BCP-ALL.

Additionally, we asked whether it was possible to identify a difference in the gene expression levels of the same AMPK-related genes that we identified through RPMA analysis among MLL-rearranged and non-translocated patients. We analyzed the gene expression profiles of 29 MLL-rearranged and 41 non-translocated pediatric BCP-ALL patients. All the patients included in this analysis were part of a larger cohort of samples analyzed by gene expression profiling during the international “Microarray Innovation in Leukemia“ (MILE) study [10]. The unsupervised analysis with the 15 probe sets corresponding to Lkb1, Ampkα and β, eNos and Bcl-2 genes was not able to accurately separate MLL-rearranged and non-translocated patients (**Figure S1A**). Moreover, when performing a comparative analysis between MLL-rearranged and non-translocated patients using the 15 probe sets of the AMPK-related genes, only one probe set (PRKAA 214917_at, corresponding to Ampkα) resulted to be differentially expressed between the two groups with a fold change more than 2.0 (**Figure S1B**). It is of note that this probe set resulted to be upregulated in the non-translocated group of patients.

In order to verify the kinase-substrate relationships within the AMPK pathway, we treated the two MLL-rearranged cell lines SEM and RS4;11 with the commercial AMPK inhibitor Compound C. As shown in **Figure 2C**, after AMPK inhibition the activation levels of AMPKα and β and all the downstream targets are markedly decreased, confirming the functional link between these proteins. In addition, apoptosis is induced in MLL-rearranged cell lines after Compound C treatment (LC50 8 µM, 48 h), while other two human non-translocated BCP-ALL cell lines are insensitive to AMPK inhibition (data not shown).

Our data provide evidence that in MLL-rearranged patients a number of directly connected kinase-substrates are activated, and this can contribute to the chemotherapy resistance observed in these patients.

### LCK Activity is Down-Modulated in Prednisone Good Responder Patients

**Figure 3:**
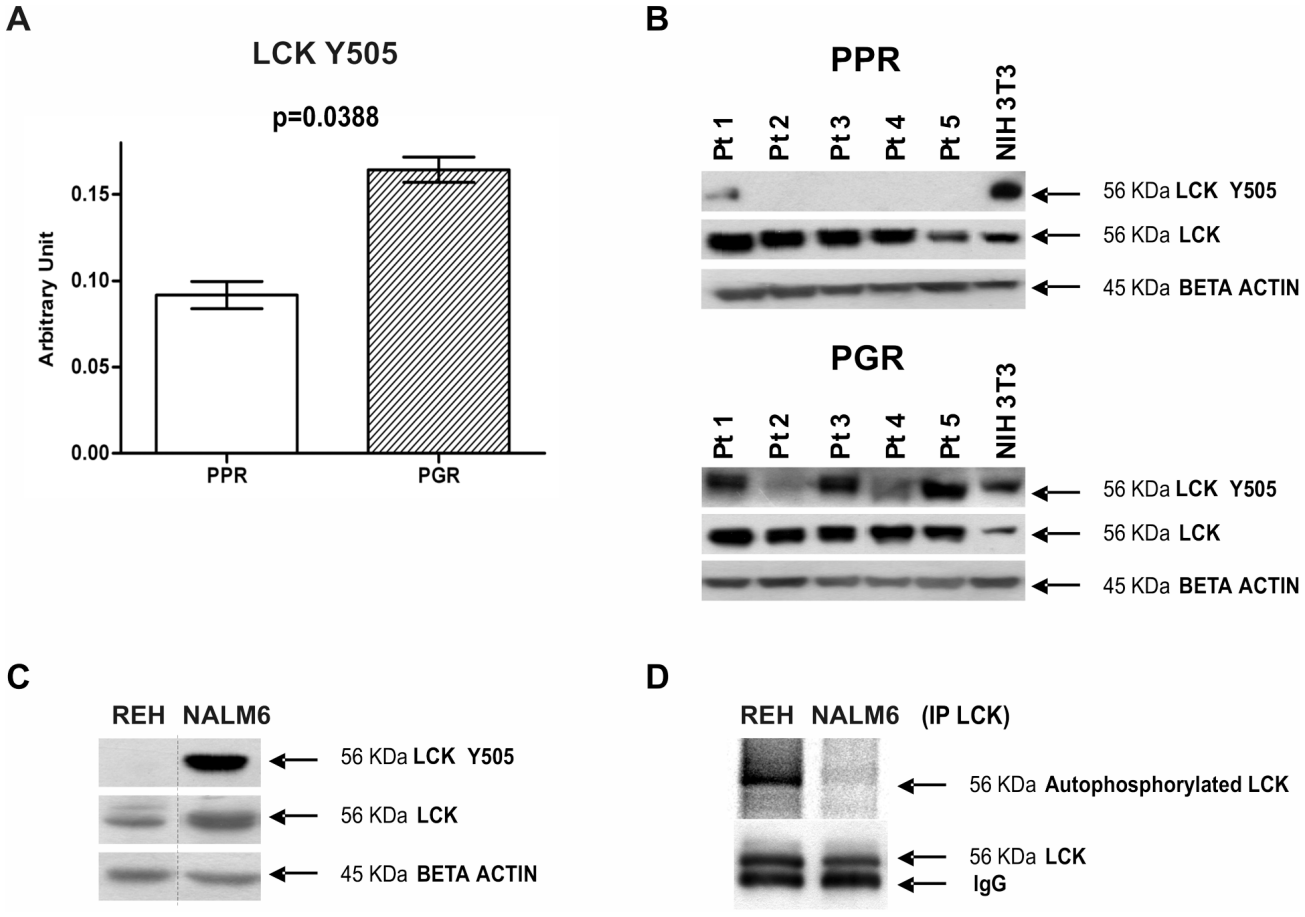
Hyperactivation of LCK in PPR patients. (A) LCK Y505 measured with RPMA is higher in Prednisone Good Responder (PGR) (0.164±0.007) than in Prednisone Poor Responder (PPR) (0.092±0.008) patients (two-sample Welch t-statistics -unequal variances- with Bonferroni multiplicity corrections p = 0.0388). (B) LCK hyperphosphorylation at Y505 in PGR patients was confirmed by Western Blot in an independent set of specimens (pediatric BCP-ALL at diagnosis: patients 1–5 are Prednisone Good Responders, patients 6–10 are Prednisone Poor Responders). NIH3T3 commercial cell lysate (BD Biosciences) was used as positive control for antibody staining. Expression of the total form of LCK does not differ between the patients. (C) LCK activation state in the cell lines chosen as *in vitro* model. REH cell lines are a model for PPR patients, while NALM6 are a model for PPR patients. The LCK activation state was evaluated through WB. (D) In vitro LCK autophosphorylation assay. LCK was immunoprecipitated from REH and NALM6 cells, and then incubated in a phosphorylation mixture at 30°C for 40 min. Autophosphorylation of LCK was analyzed by digital autoradiography on Cyclone Plus (upper gel), and by WB with anti-LCK antibody to assess the LCK amount (lower gel).

We compared the phosphoproteomic profile of 9 Prednisone Poor Responder (PPR) patients vs 109 Prednisone Good Responder (PGR) patients. Statistical analyses (two-sample Welch t-statistics -unequal variances- with Bonferroni multiplicity corrections) revealed that in PGR patients the inhibited form of the kinase LCK (LCK phosphorylated at Y505) was higher than in PPR patients (p = 0.0388) (**Figure 3A**). We confirmed the overexpression of the inhibited form of LCK in PGR patients by Western Blot in an independent set of patients (**Figure 3B**).

In order to provide the biochemical evidence of an hyperactivation state of LCK in PPR cells compared to PGR cells, we performed a radioactive autophosphorylation assay of LCK. To this purpose we selected REH cells as a model for PPR patients and NALM6 cells as a model for PGR patients [11]. We first confirmed that LCK is more phosphorylated in Y505 in NALM6 cells than in REH cells (**Figure 3C**): when similar amounts of total LCK were analyzed, the LCK Y505 phosphorylation was evident almost exclusively in NALM6 cells. As expected, on the contrary, the autophosphorylation activity of immunoprecipitated LCK was significantly higher in REH cells (**Figure 3D**).

This tyrosine-kinase, important in the regulation of growth and differentiation of eukaryotic cells, resulted to be more activated in cells that do not respond to glucocorticoids treatment, and thus activation of LCK could be considered as a putative marker for prednisone resistance.

### CYCLIN E is Upregulated in Relapsed Patients

**Figure 4:**
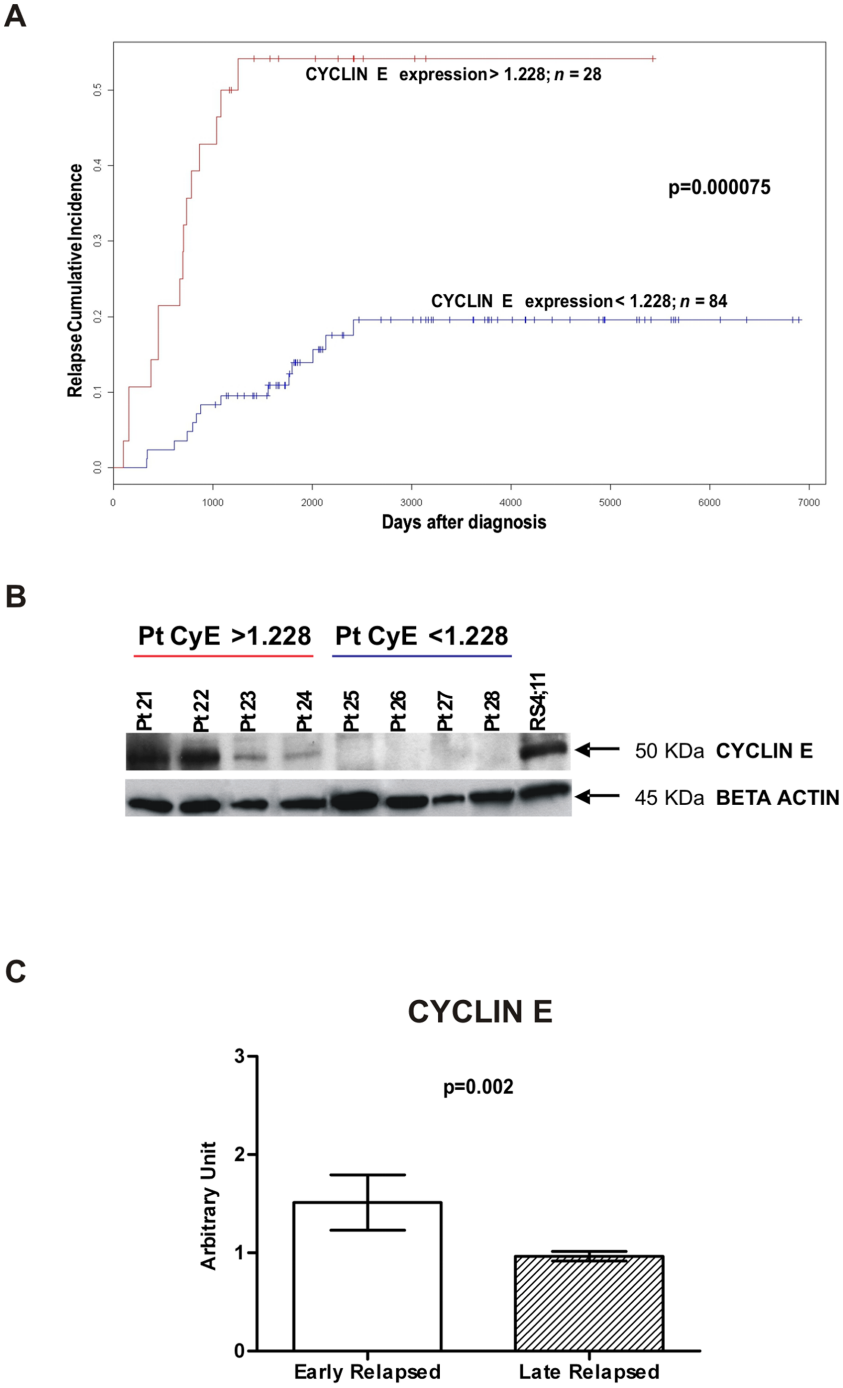
CYCLIN E expression and Relapse Cumulative Incidence. (A) Relapse Cumulative Incidence comparison between patients with CYCLIN E expression levels <1.228 (n = 84, blue line) and patients with CYCLIN E levels >1.228 (n = 28, red line). Patients with elevated CYCLIN E levels have a higher probability to relapse (CYCLIN E threshold 1.228, log rank test after Bonferroni corrections p = 0.000075). (B) RPMA results validation with Western Blot. Patients 21–24 showed high levels of CYCLIN E after RPMA analysis: 1.974, 1.896, 1.537, 1.506 respectively. Patients 25–28 showed low levels of CYCLIN E after RPMA analysis: 0.463, 0.470, 0.298, 0.292 respectively. RS4;11 cell lysate was used as positive control for antibody staining. (C) Early relapsed patients (relapsed within 910 days from diagnosis) show higher levels of CYCLIN E expression with respect to late relapsed patients (Wilcoxon test p = 0.002).

We performed a Relapse Free Survival (RFS) analysis considering patients included in AIEOP LLA 88, 91, 95 and 2000 therapy protocols in order to identify proteins related to a more aggressive disease. Relapse Cumulative Incidence were obtained through Kaplan-Meier estimates and the difference between two curves was assessed by log rank test. CYCLIN E, that regulates cell cycle steps critical for growth control, had been demonstrated to be overexpressed in many malignant tumours (for a review see Möröy et al. [12]), and in particular it could be useful to assess malignancy of blasts in adult B- and T-ALL [13]. Similarly to van Rhenen et al. [14] we thus searched for a threshold value for CYCLIN E that resulted in the largest difference in survival between the two groups defined by that threshold. We considered 10 equispaced cutoff values between the first quartile and the third quartile. A threshold was deemed valid only if the difference in the Relapse Cumulative Incidence curves was statistically significant (CYCLIN E threshold 1.228, log rank test after Bonferroni corrections p = 0.000075) (**Figure 4A**). We found that patients with CYCLIN E levels higher than 1.228 have a higher probability to relapse: 15 of 28 (53%) patients with CYCLIN E higher than 1.228 relapsed, while among the 84 patients with CYCLIN E lower than 1.228 only 14 (17%) relapsed. We confirmed RPMA CYCLIN E levels by Western Blot (**Figure 4B**). Moreover, we observed that 6 of 7 MLL-rearranged patients and 3 of 3 patients with t(9;22) are in the high CYCLIN E expression group. Furthermore, 16 of 19 patients MRD-Standard Risk have low levels of CYCLIN E, and 4 of 5 patients MRD-High Risk have high levels of CYCLIN E. In the groups determined by the above threshold we searched for differences in age, sex, immunophenotype, chromosomal translocations, DNA index, WBC count, prednisone response and MRD. From this multivariate analysis, CYCLIN E expression resulted independent from all other considered variables. We also compared CYCLIN E expression in early vs late relapsed patients. By definition, early relapsed patients are those patients who suffered of a relapse within 6 months after stopping front-line treatment, and they show a worst prognosis with respect to late relapsed patients [15]. CYCLIN E resulted significantly higher in early relapsed patients (Wilcoxon test p = 0.002) (**Figure 4C**). Because CYCLIN E in conjunction with its kinase subunit CDK2 regulates essential processes for entering into the S-phase, we also looked if differences between relapsed and non-relapsed patients could be associated to CYCLIN E together with CDK2. After Global Test analysis, the two proteins resulted to be correlated (p = 0.009395) in our patients cohort. These observations taken together strongly suggest that CYCLIN E could be considered as a marker for the aggressiveness of the disease.

## Discussion

Despite dramatic improvements in leukemia therapy over the last decades, about 20% of patients does not achieve a stable complete disease remission. In the past few months, new findings using genome-wide mutational analysis have placed central importance on protein pathway deregulation as the principal driving force in many human malignancies [2], [3]. We have originated the RPMA technology to directly quantitatively measure protein pathway function and activation to broadly map signal transduction networks and profile human cancers in order to identify groups of patients with specific molecular aberrations and to identify new targets for therapy. In this study we used the RPMA global pathway mapping technique in pediatric BCP-ALL patients to map the pathway activation status of 92 different phosphorylated and total proteins of key signalling “hubs” known to be involved in human tumourigenesis and metastasis. Correlations of signalling activation with clinical response/follow-up and known genetic information (e.g. gene rearrangements) enabled us to identify new protein pathway biomarkers that, when validated in larger clinical sets, could be used for patient stratification and targeted therapy trials.

Our first main finding concerns infants with MLL (Mixed Lineage Leukemia) rearrangements. MLL translocations are present in about 6% of pediatric leukaemia patients, especially in infants with ALL where about 75% of patients are MLL-rearranged, and their presence predicts early relapse and poor prognosis (event-free survival of 28–45%) [16], [17]. We identified a singular MLL-specific hyperactivated pathway that through AMPK phosphorylation leads to the activation of BCL-2, a well known anti-apoptotic regulator crucial for chemotherapy resistance already found to be over-expressed both at mRNA and protein levels in MLL-rearranged leukemias [18]. This pathway derangements appear to emanate from LKB1, a serine/threonine kinase that has been shown to phosphorylate AMPK [19]. AMP-activated protein kinase (AMPK) is a serine/threonine kinase that acts as a cellular fuel sensor, activated under conditions that deplete ATP and elevate AMP levels such as metabolic or environmental stresses. AMPK is known to activate endothelial nitric oxide synthase (eNOS) [20], [21] and thus to stimulate Nitric Oxide (NO) production. NO is a multifunctional transcellular messenger that can play a dual role in cancer with both pro- and anti-apoptotic effects [22], [23]. Interestingly, a prominent NO production has been observed in undifferentiated tumours [24] such as the MLL-rearranged leukemias. In addition, it has been reported that NO can prevent apoptosis by elevating BCL-2 expression both at mRNA and protein levels in B-lymphocytes [25], [26]. We showed that treatment of two MLL-rearranged cell lines with the AMPK inhibitor Compound C not only brings to AMPK deactivation, but also of its described downstream targets. This confirms the kinase-substrate relationship between these proteins, and highlights the essential contribute of AMPK in sustaining the activation of this pathway. Activation of AMPK had been studied in several tumour types because it usually leads to antitumour effects [27], but we found AMPK phosphorylation to be at the highest relative levels in the MLL-rearranged subgroup. We speculate that the role of AMPK pathway in leukemias and other hematological tumours may be different from solid epithelial malignancies, as already reported by Baumann *et al.* [28] in multiple myeloma cells. Indeed, it will be of primary interest to inhibit AMPK, and thus BCL-2, activity using commercially available AMPK inhibitors in order to more fully elucidate the functional role of AMPK activation in MLL-rearranged patients, and to evaluate AMPK as a potential new therapeutic target for this specific subgroup of patients. The mRNA results, indicating that the AMPK pathway genes are not upregulated in MLL-rearranged patients, sustain the importance to deepen with protein activation analyses in order to better define disease-related disorders in cellular metabolism, and thus to identify new molecular drug targets.

One of the strongest independent predictive factors for therapy outcome in childhood ALL is the response to initial prednisone treatment. Prednisone response is considered a backbone in Berlin-Frankfurt-Münster (BFM)-oriented protocols, and is defined by the number of peripheral leukemic blasts on day 8 of therapy [29], [30]. The threshold value for distinction between good and poor response is 1000 blasts/µl. In trial ALL-BFM 90, PGR patients showed a 6-years Event Free Survival (EFS) of 82%, while for PPR patients this was only 34% [31]. Here, we observed that in PGR patients LCK is less activated than in PPR patients (LCK Y505, p = 0.0388), and we exploited the autophosphorylation assay of LCK in order to biochemically confirm its higher activity in PPR cells. LCK is a non receptor protein-tyrosine kinase of the Src oncogene family mostly expressed in T cells, where it plays an essential role in activation and development, and in some B cells and other cancer tissues [32], [33]. Its activity is primarily regulated by phosphorylation, catalyzed by the kinase CSK, at the tyrosine residue Y505, located near the C-terminus, leading to protein deactivation [34]. In the future, if ongoing validation continues to implicate LCK activation as a predictive marker for prednisone resistance, we will investigate the causal significance of this and possibly implicate this molecule as a therapeutic target that could modulate prednisone response mechanisms. Very interestingly, inhibition of LCK through treatment with kinase inhibitors currently used in clinical practice for other indications such as BMS-354825 (Dasatinib) and STI-571 (Imatinib) [35] had already been demonstrated to be able to induce apoptosis in human T cells. Thus, it will be of interest to establish if LCK inhibitors could be useful as a possible additional support in BCP-ALL PPR patients treatment.

Our third main result is that high levels of CYCLIN E expression are indicator of a more aggressive disease. Patients who show elevated CYCLIN E expression have a higher probability to relapse. CYCLIN E had been demonstrated to be overexpressed in many malignant tumours (for a review see Möröy *et al.* [12]), and, in particular, Scuderi *et al.* [13] reported that BCP-ALL blasts of adult patients had high CYCLIN E levels and relapsed samples displayed additional accumulation of the protein. CYCLIN E regulates cell cycle progression through the restriction point R, at the end of the G1-phase, to allow cells to enter S-phase inducing S-phase specific genes. The restriction point R has been recognized to be critical for growth control and thus also for the prevention of unrestricted cell proliferation, malignant transformation and tumourigenesis. We found a cutoff value of CYCLIN E expression able to distinguish patients who have a higher probability to relapse (53% vs 17%), and this is independent from all other clinical and molecular variables. Interestingly, the elevated CYCLIN E expression group is anyhow enriched in poor prognosis patients (MLL-rearranged, t(9;22) translocated and MRD-High Risk), and CYCLIN E is more expressed in early relapsed (within 910 days from the diagnosis) patients who have a worst prognosis [15] with respect to late relapsed patients. This indicates that CYCLIN E expression could correlate with the malignant potential of the cells, and thus could be considered as a marker of the aggressiveness of the disease and a new therapeutic target in pediatric BCP-ALL.

This study emphasizes the importance of protein pathway activation mapping analysis of clinical specimens as a route for the discovery of functional derangement that may be functional, causative agents of the cancer. Proteins related to proliferation and survival such as LCK, AMPK and CYCLIN E were found to be hyperactivated or overexpressed in poor prognosis patients with BCP-ALL, and could represent new molecular drug targets in pediatric B-ALL. When further validated in functional studies, specific kinase inhibitors that target AMPK pathway, LCK-mediated signalling and CYCLIN E activation could be evaluated in prospective clinical trials whereby patients who are in need of better therapeutic options could be selected and stratified for targeted therapeutics tailored to the molecular defect.

## Materials and Methods

### Ethics Statement

The study was approved by the Ethical Committee board of the University of Padova, the Padova Academic Hospital and the Italian Association of Pediatric Onco-Hematology (AIEOP). Patient's parents or their legal guardians provided written informed consent following the tenets of the Declaration of Helsinki.

### Patients

Bone marrow samples from 118 children with newly diagnosed BCP-ALL were analyzed. Diagnosis was made according to standard cytomorphology, cytochemistry and immunophenotypic criteria [36]. The study was approved by the local ethics committees and informed consent was obtained for all patients. Samples were collected at the Pediatric Oncohematology Laboratory (Padova, Italy), between 1990 and 2006 and stored in the BioBank in liquid nitrogen in FCS+DMSO. Bone marrow mononuclear cells from patients were separated by Ficoll-Hypaque technique (Pharmacia, Uppsala, Sweden) and frozen within 3 hours after collection. The whole blood blast percentage for all samples was between 70% and 98%. Patients molecular and clinical characteristics are resumed in **Table S1**.

### Cell lines

Human leukemia cell lines SEM, RS4;11, REH and NALM6 were purchased from DSMZ German Collection of Microorganisms and Cell Cultures (Braunschweig, Germany). SEM and RS4;11 cell lines derive from BCP-ALLs carrying the t(4;11) MLL-AF4 translocation. REH cell line derives from a BCP-ALL carrying the t(12;21) TEL-AML1 translocation. NALM6 cells derive from a BCP-ALL without recurrent chromosomal translocations. Cells were cultured in RPMI 1640 (Biochrom AG, Berlin, Germany) with 10% FCS, penicillin (100U/ml) (GIBCO, Invitrogen Life Technologies, Carlsbad, CA) and streptomycin (100 µg/ml) (GIBCO), and maintained at 37°C in a humidified atmosphere with 5% CO_2_.

### Reverse Phase Protein Microarrays

#### Cell Lysis

Cells were washed with ice–cold PBS 1X and lysed on ice for 20minutes in an appropriate lysis buffer: TPER Reagent (Pierce, Rockford, IL), 300 mM NaCl, 1 mM Na orthovanadate, 200 mM PEFABLOC (AEBSF) (Roche, Basel, Switzerland), 1ug/mL Aprotinin (Sigma, St. Louis, MO), 5 mg/mL Pepstatin A (Sigma), 1 mg/mL Leupeptin (Sigma). Cell lysates were then cleared by centrifugation and supernatants were collected and assayed for protein concentration with the Coomassie Protein Assay Reagent Kit (Pierce). Cell lysates were diluted to 1 mg/ml in a mixture of 2X Tris-Glycine SDS Sample Buffer (Invitrogen Life Technologies) plus 5% of β-Mercaptoethanol. Lysates were stored at −80°C and boiled for 8minutes immediately prior to arraying.

#### RPMA Printing

Lysates were loaded into a 384-well plate and serially diluted with lysis buffer into four-point dilution curves ranging from undiluted to 1∶8. As positive controls for antibody staining we added also 3 commercial cell line lysates: A431+EGF, Hela+Pervanadate and Jurkat Apoptotic cell lysates (BD Biosciences, Franklin Lakes, NJ). We divided the 118 samples in 2 set of arrays, thus 59 and 59 samples were printed in duplicate in each array set onto nitrocellulose-coated slides (FAST slides, Whatman Schleicher & Schuell, Florham Park, NJ) with the 2470 Arrayer (Aushon BioSystems, Burlington, MA). On each set of arrays also the above mentioned cell lines and 2 bridge samples were printed for antibody signal normalization between the 2 sets. Printed slides were stored desiccated (Drierite, Sigma) at −20°C until use.

#### RPMA Staining

Selected slides were stained with Sypro Ruby (Invitrogen Life Technologies) according to the manufacturer's instruction, in order to estimate the total protein amount of each printed sample. Before antibody staining the arrays were treated with ReBlot Plus Mild Antibody Stripping Solution (Chemicon, Temecula, CA) 1X for 15minutes at room temperature, rinsed 2 times for 5minutes in PBS 1X, and then blocked for 1 hour at room temperature in blocking solution (2gr I-Block - Tropix, Bedford, MA - and 0.1% Tween-20 in 1l of PBS 1X). Blocked arrays were stained with antibodies on an automated slide stainer (Dako Autostainer Plus, Dako Cytomation, Carpinteria, CA) using the CSA kit (Dako Cytomation) as described previously [6]. Slides were air dried and scanned on a PowerLook 1000 flatbed scanner (UMAX, Dallas, TX) at 600dpi. For an example of antibody-stained slides please see **Figure S2.**

For the complete list of the 92 stained antibodies with RPMA, please see **Table S2**. Each antibody was previously subjected to extensive validation for single band specificity by Western Blot (WB). For phospho-specific antibodies, each antibody was checked for specificity using cell extracts with and without appropriate ligand induction. The 92 antibodies used in this study were carefully selected based on both their extensive validation for specificity as well as detecting key signalling molecules known for their involvement in motility, invasion, pro-survival, and growth factor signalling.

#### Image Analysis

The TIF images of antibody- and Sypro Ruby-stained slides were analyzed using Microvigene Software (VigeneTech Inc, Boston, MA) to extract numeric intensity values from the arrays images. Briefly this software, specifically developed for RPMA analysis, localizes the spots and subtracts the local background, calculating pixel intensity for each spot. The software calculates these values in the antibody-stained slides, the corresponding negative control slides (secondary antibody alone) and the total protein slide. Then, for each sample, the signal of the negative control array is subtracted from the antibody slide signal and then the resulting value is normalized to the total protein value, to ensure that intensity values were not dependent on changes in concentration of printed lysate. The data values were normalized to either one of the bridge cases to facilitate comparison of sample values between paired arrays stained with the same antibody. The data processing generates a normalized single value for each protein measured for each sample.

### Western Blot

The following antibodies were used for WB at the concentrations reported in parentheses. Primary antibodies: anti-AMPKα (23A3) (1∶1000), anti-phospho-AMPKα S485 (1∶1000), anti-AMPKß1 (1∶1000), anti-phospho-AMPKα T172 (1∶1000), anti-AMPKß1 (1∶1000), anti-phospho-AMPKß S108 (1∶1000), anti-BCL-2 (1∶1000), anti-phospho-BCL-2 S70 (1∶1000), anti-LCK (L22B1) (1∶1000), anti-LCK Y505 (1∶500), anti-β-ACTIN (1∶1000) (all from Cell Signaling Technology, Inc, Danvers, MA), CYCLIN E (BD Biosciences) (1∶500), anti-eNOS/NOS III CT (1∶1000), anti-phospho-eNOS/NOS III S116 (both from Upstate – Millipore, Billerica, MA) (1∶1000). Secondary antibodies: HRP-Goat anti-rabbit and anti-mouse IgG-conjugate (Zymed Laboratories, Inc., South San Francisco, CA) (1∶50000). Total cell lysates were analyzed by SDS-PAGE under reducing conditions, and transferred to a nitrocellulose sheet (Hybond-P, GE Healthcare, Chalfont St. Giles, UK) following standard methods. Membranes were saturated for 3 hours with 2% Amersham ECL Advance Blocking Reagent (GE Healthcare), primary antibodies were incubated overnight at 4°C and secondary antibodies for 1 hour at room temperature. The immunoreactivity was determined by an enhanced chemiluminescent reaction (Amersham ECL ADVANCE Western Blotting Detection Kit, GE Healthcare). For the stripping, membranes were incubated for 30minutes in constant rocking in a solution 25 mM Glycin, 1% SDS and pH2, then washed in T-PBS 1X and resaturated.

### AMPK inhibition in MLL-rearranged cell lines

AMPK was specifically inhibited in SEM and RS4;11 cell lines using Compound C (Calbiochem, Darmstadt, Germany) 8 µM for 48 hours. Proteins were extracted from treated and control cells as described for RPMA. Of note, in order to carefully determine the activation level of AMPKα, in these experiments we measured the phosphorylation of the main activation site that is the T172.

### Immunoprecipitation and *in vitro* kinase assay

REH and NALM6 cell lines were chosen as *in vitro* model for PPR and PGR patients respectively. Cells were lysed in a buffer containing 20 mM Tris-HCl, pH 7.5, 150 mM NaCl, 2 mM EDTA, 2 mM EGTA, 0.5% (v/v) Triton X-100, 2 mM dithiothreitol, protease inhibitor cocktail Complete (Roche), 10 mM NaF, 1 µM okadaic acid, 1 mM Na orthovanadate. LCK was immunoprecipitated with limiting amount (20 ng) of anti-LCK 3A5 antibody (Santa Cruz Biotechnology, Inc., Santa Cruz, CA), for 4 h at 4°C, followed by addition of protein A-Sepharose (Sigma). 100 µM Na orthovanadate was present throughout the incubations. Immunoprecipitates were washed once with NET buffer (50 mM Tris–HCl pH 8.0, 150 mM NaCl, 5 mM EDTA, 0.05% (v/v) Nonidet P-40, 2 mg/ml bovine serum albumin) and twice with 50 mM Tris–HCl pH 7.5, then incubated at 30°C for 40minutes with a phosphorylation mixture containing 50 mM Tris-HCl, pH 7.5, 10 mM MgCl_2_, 10 mM MnCl_2_ 10 µM [γ-^33^P]ATP (specific radioactivity ∼5000cpm/pmol) and 50 µM Na orthovanadate, in a total volume of 20 ml. Samples were then boiled for 5minutes, loaded onto 11% SDS–PAGE followed by blotting to Immobilon-P membranes (Millipore); autophosphorylation of LCK was analyzed by digital autoradiography on Cyclone Plus (PerkinElmer, Waltham, MA) to detect the radioactivity and by WB with anti-LCK to assess the LCK amount.

### Statistical Analysis

Statistical analyses were performed with R. Identification of activated proteins was obtained through Wilcoxon tests or two-sample Welch t-tests implemented in multtest package [37]. Pearson's chi-squared test was used for clinical variables. Pathways were identified using global test [38]. P-values were corrected for multiplicity using Benjamini-Hochberg method to control false discovery rate or with Bonferroni method to control family wise error rate; therefore the reported p-values are adjusted p-values. Survival curves were obtained through Kaplan-Meier estimates and the difference between two curves was assessed by log rank test. Finally, a heatmap was generated to highlight the relationships between clustering and protein expression levels.

## Supporting Information

Figure S1 AMPK-related genes are not upregulated in MLL-rearranged patients. (A) Heatmap generated with Partek Genomics Suite software using the probe sets corresponding to Lkb1, Ampkα and β, eNos and Bcl-2 genes. The unsupervised analysis is not able to accurately separate MLL-rearranged and non-translocated patients. MLL-patients are highlighted in red. (B) Dot Plot representing the raw expression data of PRKAA probe set (214917_at). The comparative analysis, performed with Partek Genomics Suite software, between MLL-rearranged (red) and non-translocated (blue) patients, using the 15 probe sets of the AMPK-related genes, shows only one probe set (PRKAA, corresponding to Ampkα) differentially expressed between the two groups with a fold change more than 2.0. AMPKα results upregulated in the non-translocated patients. Each dot represents a patient, and the boxes represent the median expression values for each group of specimens. The expression values on y axis are reported on log2 scale.
(9.54 MB TIF)

Figure S2 Example of RPMA stained slides. Slides in the picture are stained with Lck Y505 antibody. Each patient lysate was printed in a four-point dilution curve ranging from undiluted to 1∶8 in duplicate (an example is framed in red). Samples were divided in 2 set of arrays, thus 59 and 59 samples were printed in duplicate in each array set onto nitrocellulose-coated slides. As positive controls for antibody staining, in the right lower part of the slides we added 3 commercial cell line lysates: A431+EGF, Hela+Pervanadate and Jurkat Apoptotic cell lysates. On each set of arrays the above mentioned cell lines and 2 bridge samples were used for antibody signal normalization between the 2 sets.
(7.47 MB TIF)

Table S1 Patients clinical and molecular characteristics.
(0.05 MB DOC)

Table S2 Primary antibodies for RPMA staining.
(0.12 MB DOC)
