# Supplementary figures and images for: Functional Protein Network Activation Mapping Reveals New Potential Molecular Drug Targets for Poor Prognosis Pediatric BCP-ALL
Source: PLoS One. 2010 Oct 21;5(10):e13552. doi: 10.1371/journal.pone.0013552 (PMC2958847; doi:10.1371/journal.pone.0013552)

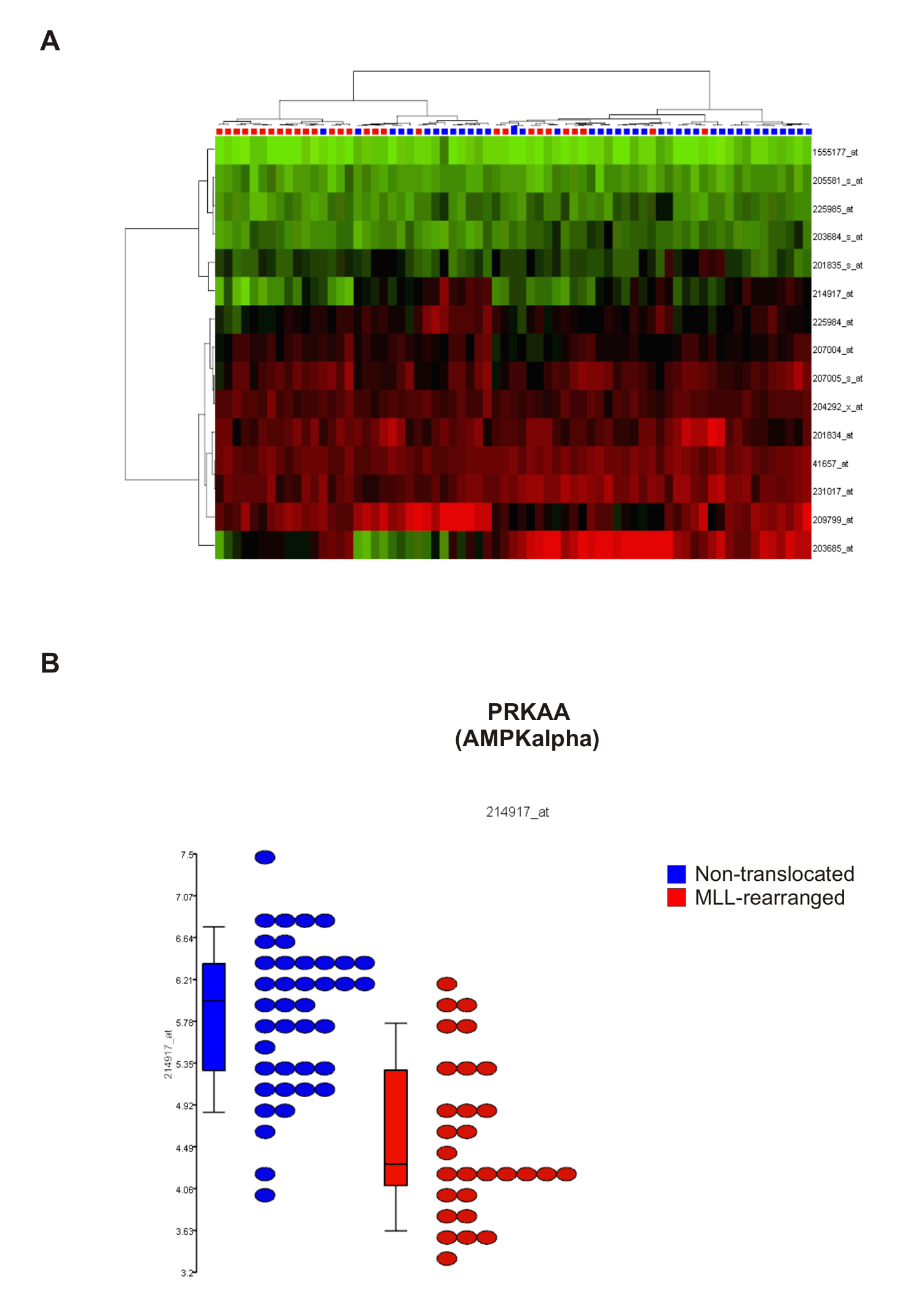

Supplement: Figure S1 — AMPK-related genes are not upregulated in MLL-rearranged patients. (A) Heatmap generated with Partek Genomics Suite software using the probe sets corresponding to Lkb1, Ampkα and β, eNos and Bcl-2 genes. The unsupervised analysis is not able to accurately separate MLL-rearranged and non-translocated patients. MLL-patients are highlighted in red. (B) Dot Plot representing the raw expression data of PRKAA probe set (214917_at). The comparative analysis, performed with Partek Genomics Suite software, between MLL-rearranged (red) and non-translocated (blue) patients, using the 15 probe sets of the AMPK-related genes, shows only one probe set (PRKAA, corresponding to Ampkα) differentially expressed between the two groups with a fold change more than 2.0. AMPKα results upregulated in the non-translocated patients. Each dot represents a patient, and the boxes represent the median expression values for each group of specimens. The expression values on y axis are reported on log2 scale. (9.54 MB TIF) [file pone.0013552.s001.tif]

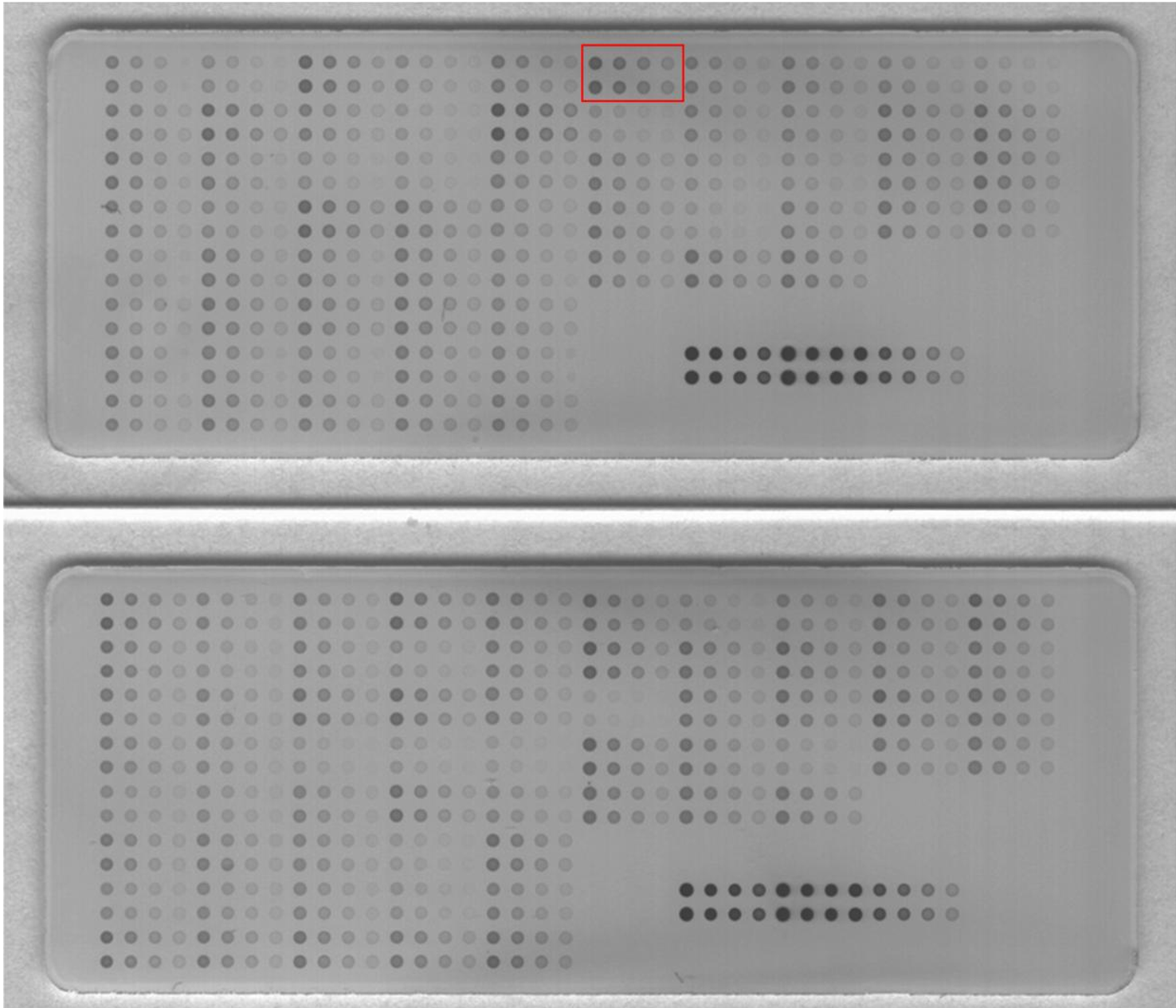

Supplement: Figure S2 — Example of RPMA stained slides. Slides in the picture are stained with Lck Y505 antibody. Each patient lysate was printed in a four-point dilution curve ranging from undiluted to 1∶8 in duplicate (an example is framed in red). Samples were divided in 2 set of arrays, thus 59 and 59 samples were printed in duplicate in each array set onto nitrocellulose-coated slides. As positive controls for antibody staining, in the right lower part of the slides we added 3 commercial cell line lysates: A431+EGF, Hela+Pervanadate and Jurkat Apoptotic cell lysates. On each set of arrays the above mentioned cell lines and 2 bridge samples were used for antibody signal normalization between the 2 sets. (7.47 MB TIF) [file pone.0013552.s002.tif]
