# Supplementary material for: Functional Protein Network Activation Mapping Reveals New Potential Molecular Drug Targets for Poor Prognosis Pediatric BCP-ALL
Source: PLoS One. 2010 Oct 21;5(10):e13552. doi: 10.1371/journal.pone.0013552 (PMC2958847; doi:10.1371/journal.pone.0013552)
Supplement: Table S1 — Patients clinical and molecular characteristics. (0.05 MB DOC) [file pone.0013552.s003.doc]

**Table S1.** Patients clinical and molecular characteristics.

| ***Characteristic*** | ***Number (%)*** |
| --- | --- |
| **Sex** |  |
| Male | 62 (52.5) |
| Female | 56 (47.5) |
| **Age (years)** |  |
| <1 | 6 (5.1) |
| 1-9 | 92 (78.0) |
| >9 | 20 (16.9) |
| **AIEOP^a^ therapy protocol** |  |
| LLA**^b^** 88 | 1 (0.8) |
| LLA 91 | 16 (13.6) |
| LLA 95 | 35 (29.6) |
| LLA 2000 | 62 (52.5) |
| Interfant | 4 (3.4) |
| **Immunophenotype** |  |
| Prepre B | 10 (8.5) |
| preB | 30 (25.4) |
| CALL | 76 (64.4) |
| Prepre B/CALL | 2 (1.7) |
| **WBC Count** |  |
| >50000 | 34 (28.8) |
| <50000 | 84 (71.2) |
| **DNA Index** |  |
| 1-1.16 | 88 (74.6) |
| >1.16 | 19 (16.1) |
| ND**^c^** | 11 (9.3) |
| **Prednisone Response** |  |
| Good | 109 (92.4) |
| Poor | 9 (7.6) |
| **Chromosome Translocation** |  |
| Neg^d^ | 36 (30.5) |
| t(12;21) | 21 (17.8) |
| t(1;19) | 7 (5.9) |
| t(9;22) | 3 (2.5) |
| MLL rearrangements | 8 (6.8) |
| ND | 43 (36.5) |
| **MRD (only AIEOP LLA 2000 patients)** |  |
| Standard Risk | 19 (30.6) |
| Medium Risk | 38 (61.3) |
| High Risk | 5 (8.1) |
| **Outcome** |  |
| Relapsed | 32 (27.1) |
| Dead | 15 (12.7) |
| Complete Remission | 71 (60.2) |

^a^AIEOP= Italian Association of Pediatric Oncohematology. ^b^LLA= Acute Lymphoblastic Leukemia. ^c^ND= No Data. ^d^Neg= negative for MLL-rearrangements, 12;21, 1;19 and 9;22.
