# Supplementary material for: Functional Protein Network Activation Mapping Reveals New Potential Molecular Drug Targets for Poor Prognosis Pediatric BCP-ALL
Source: PLoS One. 2010 Oct 21;5(10):e13552. doi: 10.1371/journal.pone.0013552 (PMC2958847; doi:10.1371/journal.pone.0013552)
Supplement: Table S2 — Primary antibodies for RPMA staining. (0.12 MB DOC) [file pone.0013552.s004.doc]

**Table S2.** Primary antibodies for RPMA staining.

| ***Antibody*** | ***Company*** | ***RPMA Dilution*** |
| --- | --- | --- |
| 14_3_3 | Upstate Biotechnology Inc. | 1:20000 |
| 4EBP1 S65 | Cell Signaling Technology, Inc. | 1:100 |
| AKT S473 | Cell Signaling Technology, Inc | 1:100 |
| AKT T308 | Cell Signaling Technology, Inc | 1:100 |
| AMPKα S485 | Cell Signaling Technology, Inc | 1:50 |
| AMPKβ S108 | Cell Signaling Technology, Inc | 1:50 |
| Annexin 1 | BD Transduction Laboratories | 1:5000 |
| Annexin 2 | BD Transduction Laboratories | 1:500 |
| Ask1 S83 | Cell Signaling Technology, Inc | 1:50 |
| ATF2 T71 | Cell Signaling Technology, Inc | 1:100 |
| Bad S112 | Cell Signaling Technology, Inc | 1:100 |
| Bad S136 | Cell Signaling Technology, Inc | 1:50 |
| Bad S155 | Cell Signaling Technology, Inc | 1:100 |
| Bak | Cell Signaling Technology, Inc | 1:50 |
| Bax | Cell Signaling Technology, Inc | 1:250 |
| Bcl-2 S70 | Cell Signaling Technology, Inc | 1:250 |
| Bcl-xL | Cell Signaling Technology, Inc | 1:500 |
| Cdk2 | Cell Signaling Technology, Inc | 1:100 |
| Chk1 S345 | Cell Signaling Technology, Inc | 1:50 |
| Chk2 S33/35 | Cell Signaling Technology, Inc | 1:50 |
| c-Kit Y703 | Zymed Laboratories, Inc. | 1:500 |
| Cleaved Casp 3 D175 | Cell Signaling Technology, Inc | 1:50 |
| Cleaved Casp 6 D162 | Cell Signaling Technology, Inc | 1:50 |
| Cleaved Casp 7 D198 | Cell Signaling Technology, Inc | 1:1000 |
| Cleaved Casp 9 D315 | Cell Signaling Technology, Inc | 1:250 |
| Cleaved Casp 9 D330 | Cell Signaling Technology, Inc | 1:50 |
| Cleaved PARP D214 | Cell Signaling Technology, Inc | 1:100 |
| CREB S133 | Cell Signaling Technology, Inc | 1:100 |
| Cyclin A | Cell Signaling Technology, Inc | 1:20 |
| Cyclin D1 | BD Transduction Laboratories | 1:20 |
| Cyclin E | BD Transduction Laboratories | 1:100 |
| EGFR Y1068 | Cell Signaling Technology, Inc | 1:50 |
| EGFR Y1148 | Cell Signaling Technology, Inc | 1:200 |
| EGFR Y992 | Cell Signaling Technology, Inc | 1:100 |
| eIF4G S1108 | Cell Signaling Technology, Inc | 1:1000 |
| Elk1 S383 | Cell Signaling Technology, Inc | 1:100 |
| eNOS-eNOSIII S116 | Upstate Biotechnology Inc. | 1:500 |
| ERK1/2 T202/Y204 | Cell Signaling Technology, Inc | 1:2000 |
| Estrogen Rec α S118 | Cell Signaling Technology, Inc | 1:1000 |
| FADD S194 | Cell Signaling Technology, Inc | 1:250 |
| FAK Y397 | BD Transduction Laboratories | 1:50 |
| FHKR S256 | Cell Signaling Technology, Inc | 1:50 |
| FHKR/L1 T24/T32 | Cell Signaling Technology, Inc | 1:200 |
| Grb2 | Cell Signaling Technology, Inc | 1:1000 |
| GSK3αβ S21-9 | Cell Signaling Technology, Inc | 1:100 |
| GSK3αβ S279/216 | BioSource | 1:500 |
| HIF1α | BD Transduction Laboratories | 1:20 |
| HSP70 | Stressgen | 1:200 |
| IKBα S32/36 | BD Transduction Laboratories | 1:50 |
| IRS-1 S612 | Cell Signaling Technology, Inc | 1:100 |
| Jak1 Y1022/1023 | Cell Signaling Technology, Inc | 1:100 |
| Lck Y505 | Cell Signaling Technology, Inc | 1:500 |
| LKB1 S428 | Cell Signaling Technology, Inc | 1:100 |
| MARCKS S152/156 | Cell Signaling Technology, Inc | 1:50 |
| MEK1/2 S217/221 | Cell Signaling Technology, Inc | 1:400 |
| MSK1 S360 | Cell Signaling Technology, Inc | 1:50 |
| mTOR S2448 | Cell Signaling Technology, Inc | 1:100 |
| p27 Kip1 | BD Transduction Laboratories | 1:100 |
| p38 MAPK T180/Y182 | Cell Signaling Technology, Inc | 1:100 |
| p70 S6 T412 | Upstate Biotechnology Inc. | 1:500 |
| p70 S6 T389 | Cell Signaling Technology, Inc | 1:20 |
| p90 RSK S380 | Cell Signaling Technology, Inc | 1:400 |
| PAK1 S199/204-PAK2 S192/197 | Cell Signaling Technology, Inc | 1:50 |
| PDGFRβ Y716 | Upstate Biotechnology Inc. | 1:250 |
| PDK1 S241 | Cell Signaling Technology, Inc | 1:200 |
| PKA C T197 | Cell Signaling Technology, Inc | 1:200 |
| PKCα S657 | Upstate Biotechnology Inc. | 1:2000 |
| PKCαβII T638/641 | Cell Signaling Technology, Inc | 1:100 |
| PKCδ T505 | Cell Signaling Technology, Inc | 1:100 |
| PKCθ T538 | Cell Signaling Technology, Inc | 1:100 |
| PKCζ/λ T410/403 | Cell Signaling Technology, Inc | 1:50 |
| PRAS40 T246 | BioSource | 1:3000 |
| PTEN S389 | Cell Signaling Technology, Inc | 1:500 |
| PYK2 Y408 | Cell Signaling Technology, Inc | 1:200 |
| A-Raf S299 | Cell Signaling Technology, Inc | 1:50 |
| B-Raf S445 | Cell Signaling Technology, Inc | 1:50 |
| C-Raf S338 | Cell Signaling Technology, Inc | 1:200 |
| Ras-GRF1 S916 | Cell Signaling Technology, Inc | 1:50 |
| S6 Ribosomal Protein S235/236 | Cell Signaling Technology, Inc | 1:200 |
| SAPK/JNK T183/Y182 | Cell Signaling Technology, Inc | 1:200 |
| SGK1 | Upstate Biotechnology Inc. | 1:200 |
| Shc Y317 | Cell Signaling Technology, Inc | 1:250 |
| SHIP Y1020 | Cell Signaling Technology, Inc | 1:50 |
| Smad2 S465/467 | Cell Signaling Technology, Inc | 1:250 |
| Src Y416 | Cell Signaling Technology, Inc | 1:100 |
| Src Y527 | Cell Signaling Technology, Inc | 1:500 |
| STAT1 Y701 | Cell Signaling Technology, Inc | 1:50 |
| STAT3 S727 | Cell Signaling Technology, Inc | 1:200 |
| STAT3 Y705 | Upstate Biotechnology Inc. | 1:100 |
| STAT5 Y694 | Cell Signaling Technology, Inc | 1:50 |
| STAT6 Y641 | Cell Signaling Technology, Inc | 1:100 |
| Tuberin-TSC2 Y157 | Cell Signaling Technology, Inc | 1:50 |
